# Knitting Mochilas: A Sociocultural, Developmental Practice in Arhuaco Indigenous Communities

**DOI:** 10.5964/ejop.v12i2.1039

**Published:** 2016-05-31

**Authors:** Lilian Patricia Rodríguez-Burgos, Jennifer Rodríguez-Castro, Sandra Milena Bojacá-Rodríguez, Dwrya Elena Izquierdo-Martínez, Allain Alexander Amórtegui-Lozano, Miguel Angel Prieto-Castellanos

**Affiliations:** a Universidad del Valle, Cali, Colombia; b Universidad de La Sabana, Chía, Colombia; Aalborg University, Aalborg, Denmark

**Keywords:** development, culture, daily practices, mochilas, knitting, childhood, Colombia

## Abstract

The purpose of this article is to analyze the psycho-cultural processes involved in knitting “mochilas” (traditional bags), a common craft in the Arhuaco indigenous community located in the Sierra Nevada de Santa Marta, Colombia. The article is structured in three parts, as follows: first, issues related to child development are discussed; then, the analysis method used to study the processes involved in the practice of knitting is presented and, finally, we reflect on the importance of recovering the sense and meaning of this everyday practice as a way to study child development.

## Introduction

### Perspectives on Human Development

The study of development has been undertaken from different perspectives by biologists, nativists and culturalists, among others, which have undoubtedly contributed to the understanding and explanation of the processes associated with the change and emergence of cognitive, emotional and social skills in children. Nevertheless, these approaches have generated fragmentation in psychology because they tended to consider development from a limited point of view or disconnected perspectives (Lewis, 2000).

The research presented here recognizes and values these different perspectives used to study human development but also aims to achieve a more integrative understanding. We approach child development from a perspective centered on processes, scenarios, paths and players that contribute to the strengthening and emergence of new skills in childhood and adolescence (Lyra, 2010; Rodríguez-Burgos, Díaz-Posada, Rodríguez-Castro, Izquierdo-Martínez, & Nassar-Pinzón, 2014). Therefore, our work is closer to a cultural perspective as we intend to analyze how the daily practices that different communities perform contribute to emergence and strengthening of skills in childhood and adolescence (Valsiner, 2012; Vygotsky, 1978).

In connection with the aforementioned perspective, the work of Silva, Correa-Chávez, and Rogoff (2010) is of great interest in the context of this paper since they have worked extensively with members of indigenous communities in Mexico and, inspired by the ideas of Vygotsky, they focused their interest on showing how children can learn and develop certain skills without necessarily going through processes of formal education (for instance, in school). More specifically, they advocated for a view of children’s development as closely linked to his/her own cultural activities. The authors share the proposals, since they consider that a child is born with certain basic skills, such as perception, attention and memory. However, through interaction with peers and adults, these abilities are transformed and become stronger as the child participates in activities and processes of cultural appropriation, including the appropriation of values and norms of behavior.

Another example of research adopting this theoretical position is represented by Barbara Rogoff’s (2012) studies in Mexico, where she worked with 34 children, ages 8 to 10. Collaborative participation and learning processes were observed in the Purépecha indigenous community. The study showed that these children were involved, on a daily basis, in almost all activities of the community until the evening. During school holidays, children live with people of all ages instead of a nuclear family as it is considered that this is where children learn social norms, respect, and reciprocity, as well as different crafts such as weaving, housekeeping, and cooking. This research concluded that parents are the ones who guide these types of participation in indigenous children; children, in turn, develop more complex conceptions and ideas about what activities they like, corresponding to their age. In this manner, parents begin to involve their children in communicative and work activities.

Mejía, Keyser, and Correa (2013), who also studied indigenous children in Mexico, brought evidence, through observation and collaborative participation, of the fact that indigenous boys and girls acquire cultural knowledge as they identify the implicit rules within their community, such as 'greeting all those who come to their homes or are met on the way'. Responsible for guiding and orienting all these behaviors are both parents and other community members.

In 2010, Silva, Correa-Chávez, and Rogoff conducted a study which brought further evidence of the fact that children of indigenous communities in Mexico, Honduras and Guatemala learn through observation and participation in their own community’s activities. One of these activities is knitting. Often, girls learn to knit without someone teaching them explicitly how to do it. It almost appears as if, spontaneously, the girls just grab a needle and start knitting a bag; this could indicate strong cultural guidance, often implicit, that these girls assimilate and, in turn, appropriate when they start being engaged in this activity.

According to the above, it is considered that indigenous children have the opportunity to learn while watching ongoing events, such as watching their grandmothers and mothers as they weave, or watching their fathers and elder brothers planting or working the land. In addition to the work of Barbara Rogoff and collaborators, the research of Patricia Greenfield (2006) is of particular interest for the present study. For two decades, she studied weaving blankets and the development of creativity in the Mexican community of Chiapas, Nabenchauk. This research allowed her to analyze the changes in the main types of basic subsistence for the community, from agriculture to commerce, boosted by knowledge of making rugs, textile design, weaving existing patterns and creating new ones among weavers in the Mayan population.

In her studies, Greenfield (2004) showed how the creative processes involved in weaving blankets evolved over time and pointed to the fact that a strong family influence is evident in the processes carried out in creating the blanket, which involves interaction and mutual influence among family members, particularly when weaving is treated as a process of sharing knowledge. The research, for example, portrays a dialogue between a mother and a daughter: The girl says: “My mother is teaching me to knit blankets, she showed me the different shapes of knitting”. Then the mother answers “If I am teaching is because she has to learn, my mother thought me to knit blankets since I was little”. The women of this community proudly displayed their weaving of blankets saying: ‘it was our mothers who taught us to weave" (p. 478). The researcher concludes that blanket weaving is based on social teachings and that creativity is subject and adapted to the means of economic livelihood.

In fact, Greenfield’s research (2006) argues for how the model of an organization had been transformed from a community and creativity one into a family model and, finally, into an individual model. In this order of ideas, it went from a model of economic subsistence to a commercial one and not only the commercial model had changed, but also the creative process since it has evolved into a more independent one than previously seen by other researchers.

In addition, it is proposed that such adaptations of creative processes and types of learning have also evolved from a family model to one based on trial and error, fostered by the new economic environment (Greenfield, 2000). The same principles could be applied to other communities because of a large globalized economic change leading towards a more commercial and larger economy (Greenfield, 2004, 2006),

Hence, in this study we approach development and community craft from an auto-ethnographic perspective. From an epistemological point of view, this approach values the study of the community life, taking into account the multiple contexts the person lives in throughout his/her existence (Blanco, 2012). We will explain this approach further in the methodology section.

### Understanding Development

It is important to express that our perspective on development does not seek to answer questions related to how much the child ‘has’, in terms of ability and knowledge, or what the child lacks to ascend to another stage, and we do not study or measure skills in an experimental laboratory not do we use tests to determine where the child can be placed on a normal curve. Conversely, our perspective focuses on studying how change happens and how cognitive and emotional processes emerge in children. That is why we take our departure in a dynamic perspective of development that will now be explained.

We believe that development is a complex and dynamic process, a key feature of systems that are open in nature is characterized by the interaction between their multiple levels. This implies recognizing that the emergence of change requires organization and structural hierarchies encompassing multicellular, genetic, physiological, psychological, social and cultural levels. Development is dynamic because it takes place on different time scales that ranges from seconds, minutes, hours, and days, to years and decades (Lewis, 2000; Lewis & Stieben, 2004; Molenaar, Brown, Caile, & Smith, 2002; Molenaar & Valsiner, 2009; Valsiner, 2004; van Dijk & van Geert, 2007; van Geert, 2000).

In consequence, we start from a dynamic perspective of development that, according to the contributions of Vygotsky (1978) recognizes several things: (1) Children do not develop alone but through interactions with parents, siblings, friends and teachers which foster the children’s development; (2) Daily practices are scenarios that contribute to their development; and (3) As researchers, we must restore the fine and detailed observation of developmental phenomena in order to identify and understand how new skills arise, who participates in this development, and what processes underlie the practices of everyday life. In this sense, the words of Cynthia Rodriguez resonate with the methodological approach that underpins the present study:

"There was a time when psychology was in no hurry. Perhaps for that reason it watched well. And everyone knows that to watch well, you need three things: watch slowly, watch a lot, and above all, with pleasure". (Cintia Rodríguez, 2006, p. 9.)

### The Arhuaco Community

The Arhuaco community is geographically located on the north coast of Colombia, in the Sierra Nevada of Santa Marta. The members of this indigenous community are known as the Arhuacos. In spite of colonization^i^, the community has largely managed to maintain its own, distinctive cultural identity. Even following centuries of colonization, the Arhuaco community remains culturally one of the strongest indigenous populations in Colombia, mainly because it preserves important traditions related to spirituality, an original mother language, typical costumes, and a philosophy and worldview specific for the Arhuaco people.

The latter are related to the Laws of origin^ii^, a set of rules and values members of the community live by and teach to future generations (Gómez, 2014). The main economic activity of the Arhuacos consists of agriculture and knitting. The members of the community are peaceful and spiritual people who enjoy following their philosophy, and consider the Sierra to be the heart of the world. In relation to this, Aty Dwrya^iii^, a member of the Arhuaco community and one of the authors of this research, says:

"The Sierra is the origin, it is a gift given (Kakuserankwa) to us by God so that it can be taken care of, cultivated and protected. It’s my home, the center of my life. It is the land of my ancestors and it will be the land of my children and my children's children. It is the place where we make offerings. It is and will be the storeroom of the world and it contributes to sustaining humanity. It's a beautiful and magical place. I think it is the only place in the world where mountains, ocean and snow-capped mountains converge. For years we have been struggling to maintain our territory, even when the government wanted to build hotels here on several occasions. We are an indigenous reserve protected by gods. We have not allowed any displacement or invasions. Tourists can visit here and eat for a while, but they have to respect the area and our traditions."

### Research Objective and Question

The main objective of this article is to analyze the psycho-cultural processes involved in knitting mochilas (traditional bags) in the Arhuaco community, located in the Sierra Nevada de Santa Marta, Colombia. In practical terms, this leads us to the following general research question: How do knitting practices support the emergence of cognitive, social and emotional skills in Arhuaco girls?

## Methodological Aspects

### How Can We Study the Knitting Practice?

In order to study development, we turn to tools such as observation, interviews and the task analysis method (Pascual-Leone, 1991; Pascual-Leone & Johnson, 2004). The latter methods allow us to analyze a particular situation or activity – in this case the knitting of *mochilas* – in order to identify the processes involved in it. We adapted this method because we are more interested here in psycho-cultural processes. Therefore, we focused on both the task (making mochilas) and the person (social psychological aspects involved in the task). It is important to add that both processes are integrated within the activity system.

The first one is the task perspective – the making of mochilas. The aspects studied in relation to it are captured by questions such as:

- What does the situation or weaving practice consist of?
- What objects or materials does the activity require?
- How does the person perform the process of knitting?
- What instructions or rules are given to perform the activity properly?
- Are there distinguishable times or stages in the activity of knitting a mochila?
- How does the person perform the process of knitting?

The second approach is related to the person and the social and psychological aspects involved in the task. Relevant questions include:

- What cognitive, emotional and cultural processes are involved in the activity of knitting and how to they develop?
- Who participates in and accompanies the development of this practice?
- How does the person resolve the difficulties or impasses that arise in the process of knitting?
- What are the meanings of the practice of knitting for the person?
- How multiple players and broader historical-cultural processes influence the activity of knitting?

### Method and Participants

The methodological approach we used in our research is based on ethnography and autoethnography, which allows us know understand the life perspective of a person from ‘within’, in an emic manner (Blanco, 2012). Ellis, Adam, and Bochner (2011) describe autoethnography as a method that combines characteristics from both autobiography and ethnography, “the storyteller and scientist; the closer the reader of an ethnography comes to understanding the native’s point of view, the better the story, and the better the science” (Liamputtong, 2009, p. 152). In relation to ethnography, Angrosino (2012) defines it as the science of describing the culture of a human group, “the art and science of describing a human group: its institutions, social behaviors, material productions and and beliefs” (p. 35).

Enabling us to observe changes in the life of the participants (Marvasti, 2004; Angrosino, 2012; Gobo, 2008, as cited by Liamputtong, 2009), autoethnography can add value to cultural and indigenous studies by providing opportunities for researchers to be self-critical of their role as researchers. Accompanied visits, visual ethnography and participant stories afford the research rich data about dialogues associated with the meanings and transformation of culture and lifestyles, providing direct information about the lived reality of the person (O'Brien & Varley, 2012). Angrosino (2012), for instance, explains that the method of ethnography has three characteristics: it is a field method due to it taking place in everyday environments; it is personalized, researchers are participants and observers; and it offers multifactorial support to others research methods such us participant observation in-depth interview, group interview, life history, focus group, etc.

In our research, Dwrya is both one of the researchers of our team and a member of the Arhuaco community from the Sierra Nevada of Santa Marta. According to Gobo (2008, as cited by Liamputtong, 2009) ethnographers entering a community use participant observation, learn the social code and understand the meaning of their actions through interaction with the participants in their natural environment. From this perspective, in our research Dwrya, due to her training in psychology, is both a lead ethnographer and an auto-ethnographer (by reflecting on what she observes, knows and learns about her own community in dialogue with the other researchers).

In this study we explore this double role of researcher and participant as we try to uncover, with Dwrya’s help, and that of other members of her community, important information regarding the practice of knitting mochilas within the Arhuaco community. We are mindful in this context of both the opportunity this type of access and research offers (e.g., more direct access to participants that are not easily accessible for research, the possibility of understanding better local culture), but also the challenges inherent to it (e.g., the need for increased reflexivity in the process of research and data presentation).

### A Note on Materials

**Figure 1:**
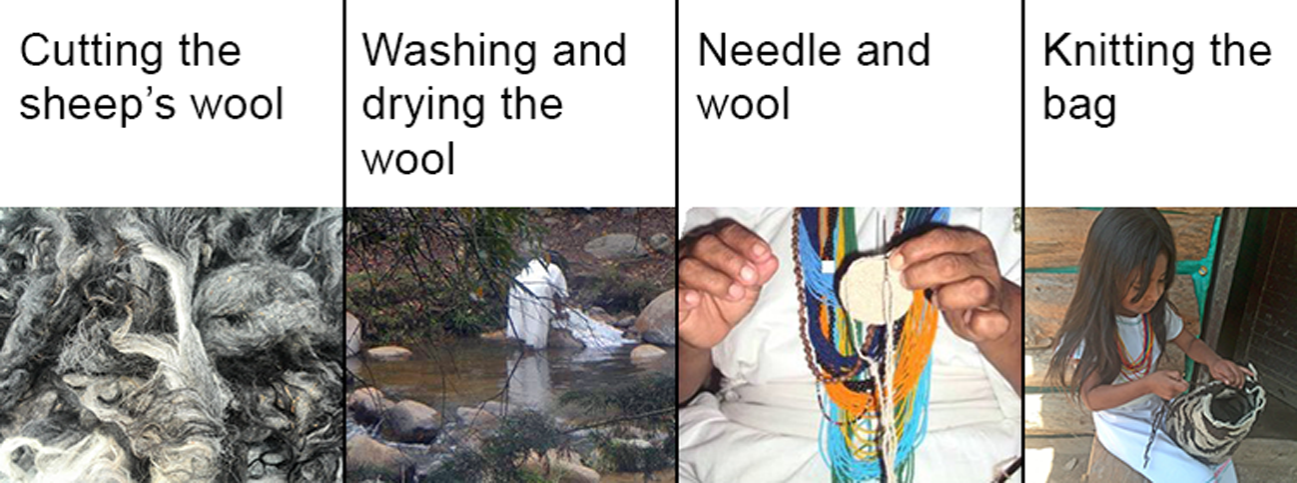
Process of mochila knit. Photos by Dwrya Izquierdo (2015).

The mochilas themselves and the setting of this activity represent the material aspect being studied. Knitting mochilas is an activity assigned exclusively to women. The materials required by the task are: sheep wool, needle and spindle. The wool is washed and dried in the sun. Subsequently, it is wound on the spindle and finally the bag begins to be knitted with the needle; this process is reflected in Figure 1. Girls aged four and older are engaged in this practice along with their grandmothers.

## Findings

**Figure 2:**
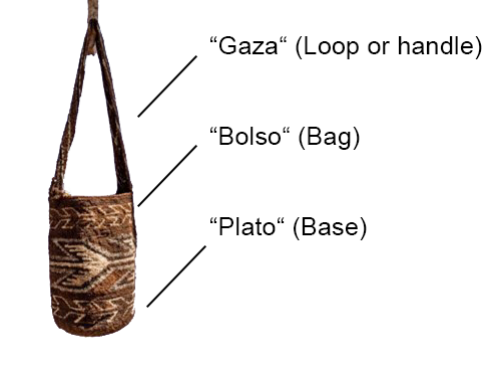
Mochila parts. Photo by Dwrya Izquierdo (2015).

**Figure 3:**
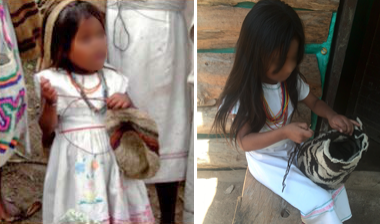
Two Arhuaco Girls Knitting. Photos by Dwrya Izquierdo (2015).

The Arhuaco women usually start knitting since early childhood (see Figure 2 for a mochila and its main parts and Figure 3 for two Arhuaco girls knitting). While many other tasks are also entrusted to them, the one seen as most important by the community is knitting because its outcomes are considered to represent thought (in its material form). The woman is the one who carries life and wisdom:

“*She is the weaver of dreams and this is the role given by the creator because she is a patient, delicate and generous dreamer. The creator has awarded her with the power to do things with her hands, to knit, which symbolizes the earthly and the divine*” (Dwrya)

### Analysis of the Mochila Knitting Practice

When analyzing this practice, we have identified four moments that are important. First, the grandmother teaches the girl the importance of knitting. Second, the girl learns about parts of the mochila. Third, the girl learns the meanings of the symbols depicted on every mochila. Last but not least, the girl knits her first mochila and offers it to the *Mamo* (the spiritual leader of the community). We briefly describe each of these stages, followed by a general analysis. Our aim in this discussion is to articulate different moments of knitting with the cognitive and social processes that emerge during the entire process.

#### First Step: Grandmother Teaches Knitting *Fique* (a Vegetal Fiber Used to Make Ropes)

**Figure 4:**
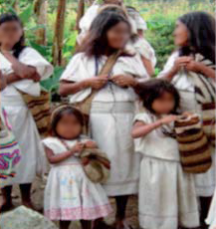
Grandmother teaches knitting fique. Photo by Dwrya Izquierdo (2015).

**Figure 5:**
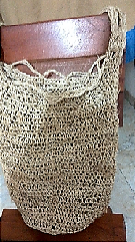
“Mochilón” knitted by an Arhuaco girl. Photo by Dwrya Izquierdo (2015).

**Figure 6:**
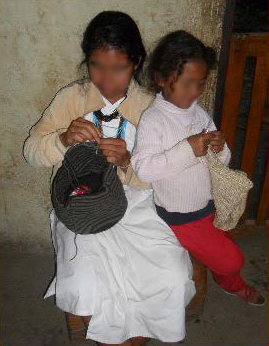
Arhuaco women and her daughter knitting one mochila and one “mochilón” respectively. Photo by Dwrya Izquierdo (2015)

*"The first thing to learn when knitting fique is that wool is removed from maguey. Grandmothers teach us to know the plant and scrape it to extract fique, then wash it, place it in the sun until it unravels and then make rows of wool from it* (Figure 4). *This thin wool can be handled very well. Grandmothers teach us how to spin fique spinning on our legs. When we have the material ready, we are taught to use the needle carefully not to sting ourselves with it. We are taught how to make thick stitches and handle the needle. Then we are shown a “mochilón” - a big and simple bag from fique* (see Figure 5) *- used to carry bananas and yucca. With the help of their grandmothers, the girls have to make big “mochilones”* (see Figure 6) *for our families, and if we demonstrate that we can make them, then we can go ahead and weave bags, not any sooner".* (Dwrya)

It is very interesting to analyze how grandmothers’ collaboration and support play an important part in being introduced, from a very young age, to the practice. Older women in the family play an active role and teach young girls how to recognize and identify the plant. It is also significant to see how the child uses her body, in this case her legs and hands, while applying the new knowledge.

Knitting involves bringing two elements together, namely the needle and the wool. The girls have to establish a relationship between the needle, wool and, in the end, the knowledge of how to make the *mochilón*. They must coordinate visual schemas, but also should simultaneously integrate what is expected of them (to knit well) and observe some restrictions, e.g., paying attention not to sting themselves and not to make wrong stitches. Various studies explain how this series of activities are related to the frontal lobe, which is closely associated with executive functions such as cognitive flexibility, choice of objectives, planning, self-control, working memory and problem solving, among others (Capilla et al., 2004; Soprano, 2003).

On the other hand, it is important to emphasize that the process of knitting involves a readiness drive that is important. This readiness implies a regular direction by mothers and grandmothers in order for the young girl to develop the ability to knit correctly. But, beyond teaching how to use the needle, what is more important is the meaning that the girl invests in the development of these special objects - bags for her family. It is an object that will have an end and the girl is not a mere spectator who observes and copies, but rather knits, creates, and brings an object into her home.

The knitting practice involves several cognitive and social processes that one cannot learn with the “naked eye”. This means that it is not easy to learn just by looking at it once, the knitting practice involves a series of abilities and dedication during a lot of time in a particular context. According to Young Shin and Soo Ha (2011), in their research about the Korean knitting practice, knitting is not only a craft production but also a form of consumption and is composed of some basic elements: purchase of materials, designing, physical labor and production of material goods (p. 105). Also, they describe knitting as a practice that implies a close relationship among the physical, mental, and emotional dimensions of the artisan. They explain these emotional effects as “the combination of simple and repetitive physical movements and control of the overall process by paying attention to regular changes within knitting patterns generate a state of high concentration” (p. 109). These rhythmic movements immediately instill a feeling of familiarity that gives people great comfort.

#### Second Step: The Bag and its Components

The production of the bag begins with cutting the goat or sheep wool, washing and drying it with its natural colors. Then follows the spinning process and bonding of fibers”.

Following that, Dwrya remembers how her grandmother usually showed one model bag (see Figure 6) and said*:*

“We are going to make stitch by stitch. First, we are going to create a background that is like knitting a circle or a plate and then continue knitting around it”.

The grandmother explains that there are five stitches, which will then be increased. Then, they will continue in Sequences 6, 7 and so on, thus giving the bag a shape. She also teaches how to alternate 5 black lines, then 5 white lines.

*"Then to give it height, we put our hand on the bottom of the plate. The bag should fit around the forearm, to see whether it is already finished. Finally, we make the handle that is of a different material similar to macramé, which is woven with a thread one side, which is attached to a piece of wool. It is weaved one on the top and the other one on the bottom. In the end, this would be the handle we're going to put on the bag. The handle can be made slightly longer or the size we want. Now we finish the handle, stick it onto the bag and then we go on to make another row. That is the cold sore, which is another larger row that remains at the top of the bag".* (Dwrya)

Certainly, counting is also part of learning, because the girls are told to make five black stitches, then return and make five white ones. It is indeed a trick used to be able to count and exercise the imagination because, while looking at other designs, the girls will make one design of their own.

At this point in the process of knitting the bag, it is important to emphasize the aesthetic details and design of the bag. The grandmother starts giving very specific instructions that will enable the child to successfully complete her bag. Here, it is important for the child to pay particular attention to two things. First, the instructions must be followed and the knitting done step-by-step according to them. Second, the child must take into account the numerical sequences and concepts for knitting that are usually very precise. As the girl is making stitch by stitch, it is explained to her that a part of the history of culture of her community is also being recorded in her knitting.

Studies, such as the one of Adamski and Borowik (2014), show that the activity of knitting and development of these skills in childhood promotes problem solving and development of fine motor skills as well as visual motor coordination, results that apply perfectly to the activity of Arhuaco girls. At the neuropsychological level, we can note from the studies of García, Enseñat, Tirapu, and Roig (2009) that there is a critical period in the development of the child within his/her first five years as it is at this time that the brain has higher growth, known as processes of myelination and synaptic pruning, which promote synaptic connections and, in turn, the development of the frontal lobe (Capilla et al., 2004). In other words, this practice builds neural plasticity, defined by Narbona and Crespo (2012) as the ability to model the nervous system’s structure and function under the influence experience, resulting in learning processes.

#### Third Step: Symbols and the Bag

Producing the bag follows a specific order. First, *mochilones* are made from *fique*, then bags from wool with stripes or single color, which are part of the basic form of knitting, and then begins the teaching of how to depict symbols that can be highly sophisticated.

**Figure 7:**
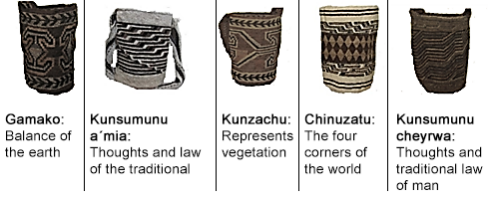
Arhuaco designs used in the weaving and knitting of the bag. Photos by Dwrya Izquierdo (2015).

*"Granny presents one design, although I think there are more than 20 designs. Each design has a meaning* (see Figure 7). *Carry bags or indigenous drawings depict animals and other objects related to the worldview and everyday life of the Arhuacos. Grandma teaches design and how it represents the elements of nature, and then she explains how the design is made. Some of the most important designs are the Gamako (frog), symbol of fertility, aku (rattlesnake), kaku Serankua (the father creator of the Sierra), gwirkunu (hills and lakes), urumu (snail), kunsamunu a'mia (Woman Thoughts), kunsamunu cheyrua (Man Thoughts), kanzachu (tree leaf), chinuzatu (four corners of the world), kambiru (scorpion's tail or scribble) and phundwas (snowy peaks of the Sierra)”.* (Dwrya)

In the final part of knitting the bag, the cultural significance is delved into, along with all the symbols represented on the bag. There are a number of drawings that can be captured on each bag, which all have a specific meaning. During knitting, they become a way of telling and remembering the meanings of the Arhuaco culture on the bags. This activity speaks of cultural memory which, according to van Dijk and van Geert (2007) is the accumulation of various elements that are telling a story over time; they begin to be appreciated as mediated memories that are part of individual identity, as well as collective identity in a community.

In research conducted by Mege (1990), everything that is expressed in the community’s weaving carries a specific intention. Colors and designs are not chosen randomly, but they depend on who they are intended for: the hierarchy of who is going to receive them. In the case of the Arhuaco community, the first bag that is knitted, for example, is intended for *Mamo*. Every bag has a different connotation and specific meaning.

#### Fourth Step: The Completed Bag, an Offering to Be Given to the Mamo

**Figure 8:**
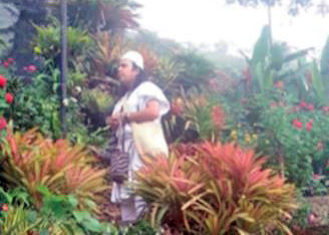
One of the Arhuaco community’s Mamos. Photo donated by Kasokaku Busintana Mestre (2014)

*"The first bag is carried to Mamo who is the spiritual leader of the indigenous community* (see Figure 8). *He grants the spiritual permission so the child can continue weaving and complying with the order and permanence of life. Since this bag is a tribute to be surrendered to the land for all the benefits received, such as water, food, air, plants, animals and all the resources that allows her to create her own spiritual and material world"* (Dwrya)

The *Mamo* is the most important authority in La Sierra Nevada, he is the owner of the traditional knowledge intended to guide the development in the life cycle of each of individuals inside the community. His role is to share and cultivate the bond between nature, the territory and communities themselves. He is considered indispensable for people to keep their own values and preserve the origin laws.

In the quote above, the *Mamo* is presented as the one who possesses the ancestral wisdom of the indigenous communities and his responsibilities as leader include being a spiritual and political leadership, medic, priest and advisor. The *Mamos* give voice to the community: “We, the natives, were located in sacred territories to take care of the spiritual equilibrium and sustainability of life in the whole dimension of the world and the universe; it is our duty to contribute with payments, through the spiritual rituals” (Gómez, 2014, p.159). The *Mamo*’s main function is to preserve the equilibrium between nature and man, but also to preserve the traditions, and defend mother earth. To do so, he performs different kinds of rituals to pay off the debt of other persons. He seems to be the coach of the community and he is the one who must share and help them, so they can take care of nature together. The Declaration to humanity of the *Mamos* in the Sierra Nevada notes:

“Sierra Nevada it is the hearth of the world, the primary and vital source of the whole world, origin of life and spiritual equilibrium of mother earth… to us, it is everything that owns a sacred spirit and must be respected. That is why everything is sacred and we feel it like part of our family: the air, the fire, the trees, the insects, the stones, the mountains… we live in a constant communication with them through our powers of knowledge and spiritual activities” (Gómez, 2014, p. 169).

In consequence, being a *Mamo* implies a huge responsibility that is entrusted on an individual since childhood. The elder *Mamo* thus decides, from early on, who is the next *Mamo*, taking in to account his physical and family features. Once he is selected, the process of teaching him the community’s traditions, history, and rituals that are transmitted from generation to generation begins. The preparation of the *Mamo* requires discipline. This depends on how he assumes the role as the biggest public authority, spiritual leader, and economic advisor of the community during his entire life.

**Figure 9:**
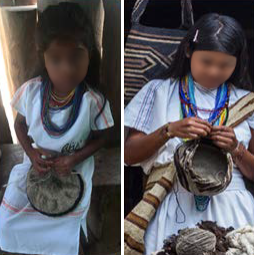
Arhuaco girl and woman knitting mochilas, respectively. Photo by Dwrya Izquierdo (2015).

About the first mochilas made by young girls (see Figure 9) it is important to note that, once they are finished, the grandmother and granddaughter take them to the *Mamo* and they put inside as a gift a load of coffee, yucca, and plantains. He receives the gift, checks the knitting and congrats the young girl for the mochila. Also he explains to the girl why it is important to make mochilas and ends this rite with a prayer.

From our perspective, the actions and words of the *Mamo* represent one of so many ways in which this community passes on its own cultural identity. Finally, it is essential to recognize the fundamental value that the mochilas have for the community due to their meaning, elaboration and direct relation with the preservation of an indigenous culture.

Besides, related to the economic aspect of the mochilas, the activity of selling them is oriented towards supplying the needs related to the home context, to buying the food, the clothes and the shoes needed. Even more, the activity of selling mochilas can be motivated by gaining popularity inside the market of crafts in Colombia (Zalabata, 2008).

On the other hand, women, as they thread the material and knit the mochila, imprint emotion, thought, traditional practices and their vision of the cosmos. This is why, the mochila, more than being just an element used by the indigenous people, is a social archive that has inscribed in its knits ancestral, social and technical practices that assign a social and economic role to women in society (Rodríguez-Burgos et al., 2014).

## Conclusion

The objective of this paper was to analyze the cognitive, social and emotional processes involved in the practice of weaving traditional bags. We started from the premise that “development is a dynamic and complex process that behaves like an open system” (Lyra, 2010; Rodríguez-Burgos et al., 2014; Valsiner, 2004). This becomes evident in the task analysis performed here (Pascual-Leone & Johnson, 2004), in which an activity of daily life facilitates the development of new skills. In that sense, researchers are invited to explore further and restore the value of daily activities for human development.

We are convinced that, in order to study development, it is not always necessary to resort to sophisticated laboratory studies. Our data show that everyday scenarios and fieldwork are extremely rich and should be the basis for further investigations. For instance, related to knitting practice we find some studies that show how knitting is like threading a story. For example, Lutton (2012) explains how, in the Waldorf School of Chicago, knitting and weaving practices are part of the curriculum: children are learning to focus and concentrate. They are gaining fine motor skills needed for writing. They see patterns. They move from left to right, the same way they read. They are gaining confidence. Besides, Riley, Corkhill, and Morris (2013), in their study of adulthood, conclude that knitters who knit frequently are calm, happy and experience higher cognitive functioning. This kind of research supports the idea that knitting offers many benefits for development. Furthermore, these researchers write about how knitting helps knitters learn about valuable life skills, such as perseverance, patience and creativity in the context of education. Some teachers are beginning to use knitting in the classroom in order to teach key life skills to their students. For instance, in Bogotá, Colombia, there is a school project called “*Tejiendo Sueños*” (Knitting dreams), where two professors teach their students different types of knitting and, through this activity, the students learn to be patient, which ultimately improves their school life (Secretaría de Educación del Distrito, 2013).

As such, future studies on the topic could focus on longitudinal research meant to capture the way these and other skills emerge by performing daily practices. This is in line with our commitment to promote practices that preserve cultural identity, studied through direct and detailed processes of observation, interviews and analysis. As such, Piaget's words are inspiring for this kind of work: *“[…] a good analysis surpasses all statistics*” (Piaget, 1936, p. 69-70)

This research contributes to the transmission of cultural knowledge in narrative terms; the narratives in this study outline the origins and meaning of knitting as an important part of the daily activity of indigenous women and cultural development within the Arhuaco community. Besides, using an ethnographic approach, we can evidence how the development of young indigenous girls is permeated by beliefs, patterns and traditional principles that shape how they act in daily life beyond the knitting of mochilas.

This investigation aims, at a broader level, to return to the Arhuaco indigenous women the acknowledgement for their work and admiration for the management of social responsibility, their contribution to the construction of identity remembering that is imprinted in the item knitted by their own hands and in the cotton and vegetable fiber they use (Rodríguez-Burgos et al., 2014).

There is also a practical side to conducting and continuing this line of research. It has been repeatedly noted that the Arhuaco community enjoys tremendous cultural richness and plenty of opportunities to scaffold the development of children, the practice of knitting being only one of these opportunities. It is important thus to study, understand, and foster such daily practices in order to support child development in indigenous communities and also gain new insights into other forms of community life and their importance for socialization and growth.
